# GLUTATHIONE, MITOCHONDRIAL DEFECTS, AND A UNIQUE METABOLIC CYCLE IN OLDER HUMANS: IMPLICATIONS FOR SARCOPENIA

**DOI:** 10.1093/geroni/igz038.956

**Published:** 2019-11-08

**Authors:** Rajagopal V Sekhar, Jean Hsu, Farook Jahoor, Shaji Chacko, Premranjan Kumar, Chun Liu

**Affiliations:** 1 Baylor College of Medicine, Houston, Texas, United States; 2 Baylor College of Medicine, Houston, United States

## Abstract

Sarcopenia in aging leads to decreased muscle mass and physical-function (muscle strength and exercise capacity), but underlying mechanisms are not well understood and effective interventions are limited. We hypothesized that deficiency of the intracellular antioxidant protein Glutathione initiates a unique self-perpetuating metabolic cycle linking impaired fasted mitochondrial fuel-oxidation (fMFO) to protein catabolism and contributes to sarcopenia. We also hypothesized that supplementing the Glutathione precursor amino-acids glycine and N-acetylcysteine (GlyNAC) to correct Glutathione deficiency in older humans could reverse these defects. We tested our hypothesis in a 24-week open-label clinical-trial in 8 older-humans (74y) studied before and 24-weeks after GlyNAC supplementation, compared to 8 gender-matched unsupplemented young-controls (25y), and measured intracellular Glutathione concentrations, fMFO, physical-function, muscle-protein breakdown-rate (MPBR), gluconeogenesis, and urine nitrogen-excretion (UNE). GlyNAC supplementation in older humans corrected Glutathione deficiency and restored impaired fMFO (to levels in young controls), lowered MPBR and UNE, and increased physical-function, but did not affect gluconeogenesis or increase lean-mass, and suggest that muscle amino-acids are utilized for energy needs rather than glucose production. The absence of an increase in lean-mass suggests that GlyNAC should be combined with anabolic agents for potential benefits in combating sarcopenia. Overall, these results indicate the presence of a unique reversible metabolic cycle in older humans initiated by Glutathione deficiency which results in impaired mitochondrial fatty-acid and glucose oxidation, muscle-protein breakdown, UNE, and leads to deficiency of glycine and cysteine which re-initiate the cycle. These data have implications for improving physical-function and muscle mass in age-associated sarcopenia, and warrants further investigation.

